# Thrombin-Activatable Fibrinolysis Inhibitor (TAFI) Deficient Mice Are Susceptible to Intracerebral Thrombosis and Ischemic Stroke

**DOI:** 10.1371/journal.pone.0011658

**Published:** 2010-07-19

**Authors:** Peter Kraft, Tobias Schwarz, Joost C. M. Meijers, Guido Stoll, Christoph Kleinschnitz

**Affiliations:** 1 Department of Neurology, University of Wuerzburg, Wuerzburg, Germany; 2 Departments of Vascular Medicine and Experimental Vascular Medicine, Academic Medical Center, University of Amsterdam, Amsterdam, The Netherlands; Maastricht University, Netherlands

## Abstract

**Background:**

Thrombus formation is a key step in the pathophysiology of acute ischemic stroke and results from the activation of the coagulation cascade. Thrombin plays a central role in this coagulation system and contributes to thrombus stability via activation of thrombin-activatable fibrinolysis inhibitor (TAFIa). TAFIa counteracts endogenous fibrinolysis at different stages and elevated TAFI levels are a risk factor for thrombotic events including ischemic stroke. Although substantial in vitro data on the influence of TAFI on the coagulation-fibrinolysis-system exist, investigations on the consequences of TAFI inhibition in animal models of cerebral ischemia are still lacking. In the present study we analyzed stroke development and post stroke functional outcome in *TAFI^-/-^* mice.

**Methodology/Principal Findings:**

*TAFI^-/-^* mice and wild-type controls were subjected to 60 min transient middle cerebral artery occlusion (tMCAO) using the intraluminal filament method. After 24 hours, functional outcome scores were assessed and infarct volumes were measured from 2,3,5-Triphenyltetrazoliumchloride (TTC)-stained brain slices. Hematoxylin and eosin (H&E) staining was used to estimate the extent of neuronal cell damage. Thrombus formation within the infarcted brain areas was analyzed by immunoblot. Infarct volumes and functional outcomes did not significantly differ between *TAFI^-/-^* mice and controls (p>0.05). Histology revealed extensive ischemic neuronal damage regularly including the cortex and the basal ganglia in both groups. TAFI deficiency also had no influence on intracerebral fibrin(ogen) formation after tMCAO.

**Conclusion:**

Our study shows that TAFI does not play a major role for thrombus formation and neuronal degeneration after ischemic brain challenge.

## Introduction

The majority of ischemic strokes are caused by thromboembolic occlusion of brain arteries [1]. Consequently, thrombolysis using recombinant tissue plasminogen activator (rt-PA) is an established therapy during the first 4.5 h after stroke onset but efficacy is only moderate at best and about 90% of all stroke patients must be excluded from rt-PA treatment due to numerous contraindications [2], [3]. Current strategies for secondary stroke prevention comprise platelet inhibitors and long-term anticoagulation with warfarin in the case of cardioembolic stroke. However, as a major drawback these substances are all associated with increased bleeding complications [4]. Hence, there is a significant unmet medical need for more effective and safer therapies in patients with acute or recurrent stroke.

Two distinct pathways of blood coagulation can initiate the formation of a fibrin thrombus, the extrinsic and the intrinsic pathway [5]. Both are activated in a “waterfall-like” manner involving a series of trypsin-like serine proteases such as the tissue factor (TF)/FVIIa complex (extrinsic pathway) or FXII (intrinsic pathway) finally culminating in the generation of thrombin [6]. Thrombin is considered a key component of blood coagulation [7]: It can initiate clotting by activating FV and FVIII, platelets, and platelet-bound FXI. Moreover, the conversion of prothrombin to thrombin by FXa and FVa further propagates coagulation. Finally, thrombin is critically involved in thrombus stabilization via thrombin-dependent FXIIIa-driven cross-linking of the fibrin network and activation of thrombin-activatable fibrinolysis inhibitor (TAFI). Activated TAFI (TAFIa) is part of a highly dynamic system that downregulates fibrinolysis. Activation occurs with high doses of thrombin in the absence of thrombomodulin in the setting of the intrinsic pathway of coagulation, or with low concentrations of thrombin in complex with thrombomodulin [8]. TAFIa acts through removing carboxy-terminal lysine residues of partially degraded fibrin, leading to elimination of plasminogen binding sites, inhibition of plasminogen activation and impaired fibrinolysis [8], [9]. Several polymorphisms in the human TAFI gene have been identified that influence TAFI antigen levels [10], [11] and elevated serum TAFI is a risk factor for deep vein thrombosis [12] and coronary artery disease [13]. In contrast, available data on the role of TAFI in cerebral ischemia are limited but its function as an endogenous fibrinolysis inhibitor and the observation that TAFI-deficient (*TAFI^-/-^*) mice do not show abnormal bleeding [14], [15] renders TAFI an attractive therapeutic target in different thromboembolic disorders including stroke. In the present study we assessed stroke development, intracerebral fibrin formation and post stroke functional outcome in *TAFI^-/-^* mice.

## Results

**Figure 1:**
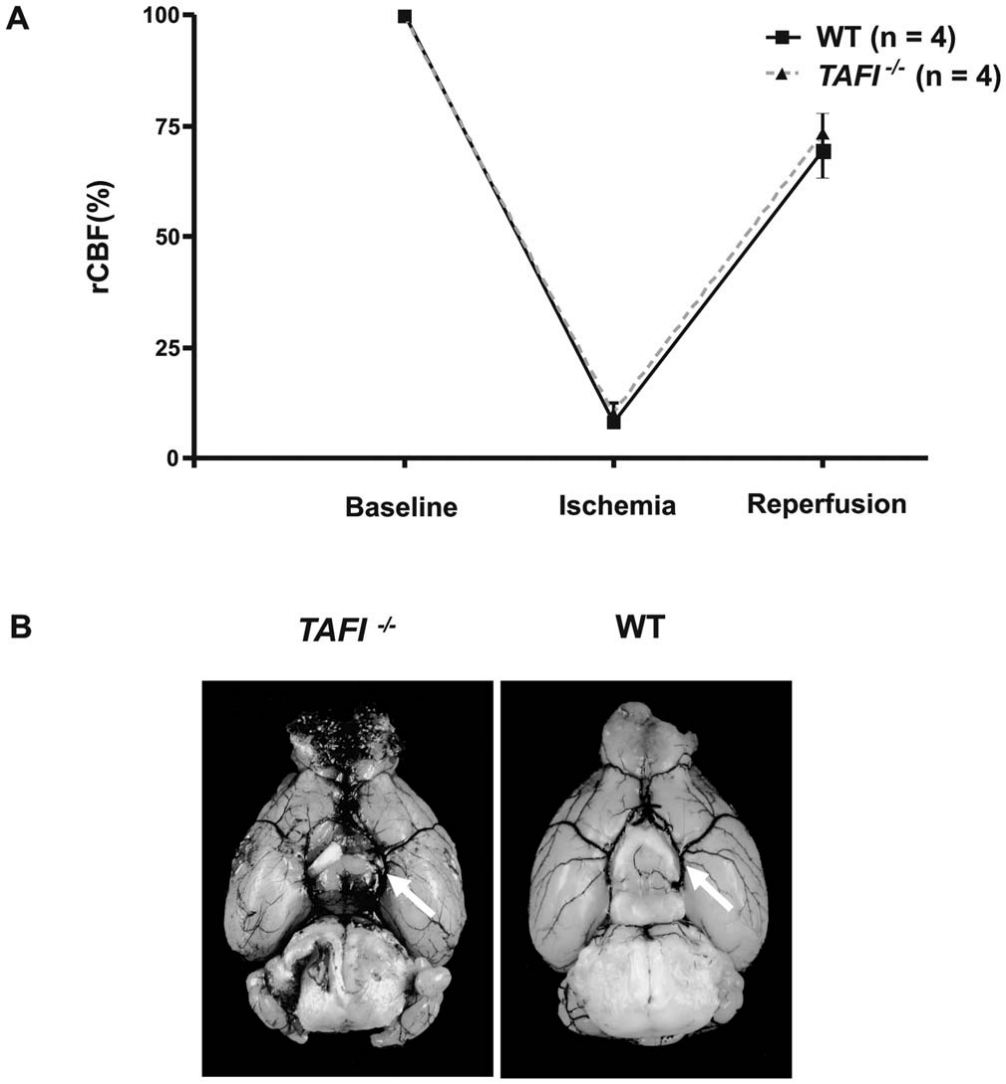
rCBF and the anatomy of the cerebral vasculature do not differ between *TAFI^-/-^* mice and controls. (A) Determination of regional cerebral blood flow (rCBF) using Laser Doppler flowmetry before the occlusion of the middle cerebral artery (baseline), 10 min after the occlusion (ischemia) and again 10 min after the removal of the filament (reperfusion) in *TAFI^-/-^* mice and wild-type controls (n = 4/group). No significant differences in rCBF were observed between the two groups. 1-way ANOVA, Bonferroni post hoc test. (B) Assessment of the cerebral vasculature in wild-type and *TAFI^-/-^* mice. A complete Circle of Willis (white arrows) was identified in all animals studied and the distribution of the MCA trunk and branch appeared to be anatomically identical among the genotypes.

The middle cerebral artery (MCA) was occluded with a silicon-coated filament for 60 min (transient middle cerebral artery occlusion, tMCAO) to induce focal cerebral ischemia in mice [16], [17]. After advancing the filament to the origin of the MCA the decrease in regional cerebral blood flow (rCBF) was similar between wild-type mice and *TAFI^-/-^* mice (8.3±4.2% vs. 10.2±1.5% of baseline levels; p>0.05) (Figure 1A). Ten minutes after removal of the filament (reperfusion) rCBF in the MCA territory was reconstituted to ∼70% of baseline levels and again did not significantly differ between the two mouse groups (69.5±8.3% vs. 73.4±10.1% of baseline levels; p>0.05) (Figure 1A). These findings exclude that *TAFI^-/-^* mice show preformed rCBF alterations related to the genotype and prove that MCA occlusion and reperfusion were sufficient in our model. Along these lines, no aberrations in the cerebral vasculature were observed in TAFI-deficient mice as assessed by transcardial ink perfusion (Figure 1B).

**Figure 2:**
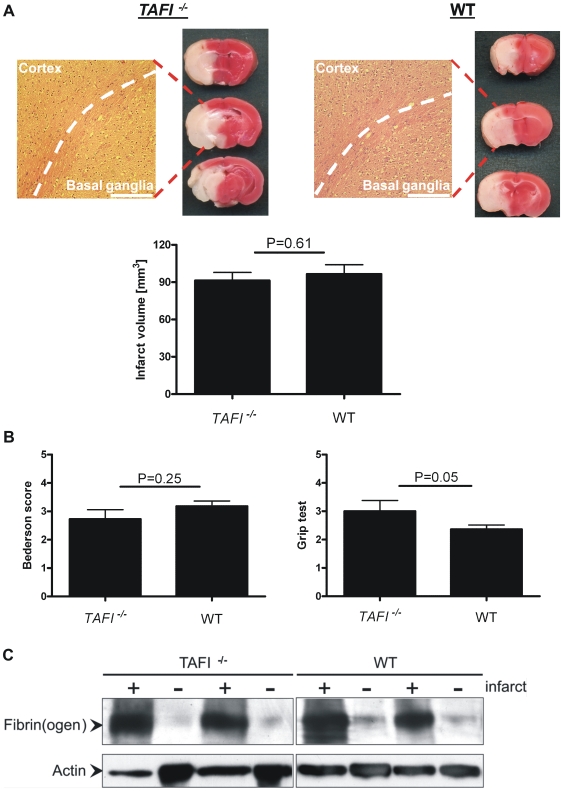
TAFI deficiency does not improve outcome after experimental stroke in mice. Infarct size and functional outcome in *TAFI^-/-^* mice and controls (WT, wild-type) 24h after 60 min transient middle cerebral artery occlusion (tMCAO). (A) (top) Representative 2,3,5-Triphenyltetrazoliumchloride (TTC)-stained coronal brain sections from the two animal groups. Ischemic infarctions appear white and regularly include the neocortex and basal ganglia as confirmed by hematoxylin and eosin (H&E) staining (bar represents 250 µm, dotted white line indicates the border between the cortex and the basal ganglia). (bottom) Infarct volumes on day 1 after tMCAO in *TAFI^-/-^* mice and controls as determined by planimetry (n = 11–12/group). Non-parametric Mann Whitney test. (B) Neurological Bederson score and grip test score on day 1 after tMCAO in *TAFI^-/-^* mice and controls. In line with the results on infarct volumes, no significant functional differences became apparent. Non-parametric Mann Whitney test (C) Accumulation of fibrin(ogen) in the infarcted (+) and contralateral (−) cortices of *TAFI^-/-^* mice and controls. Fibrin(ogen) was analyzed by immunoblotting 24 h after ischemia. Two representative immunoblots of each group are shown.

As a next step, we determined infarct sizes and the extent of neuronal damage in *TAFI^-/-^* mice and controls. 24h after tMCAO no significant differences in infarct volumes were observed between the two groups as revealed by 2,3,5-Triphenyltetrazoliumchloride (TTC) staining and successive infarct planimetry (91.4±21.5 mm^3^ vs. 95.6±27.2 mm^3^; p>0.05) (Figure 2A).

In line with these findings, H&E staining confirmed widespread ischemic neurodegeneration in both groups which regularly expanded to the basal ganglia and the neocortex (Figure 2A).

Detailed analysis of the functional status also showed no significant differences between *TAFI^-/-^* mice and controls for the grip test assessing motor function and coordination (3.0±1.3 vs. 2.4±0.5; p = 0.05) and for the Bederson score assessing global neurological function (2.7±1.1 vs. 3.2±0.6; p = 0.25) (Figure 2B). Finally, thrombus formation, i.e. deposition of fibrin(ogen) within the infarcted cortices of *TAFI^-/-^* mice and wild-type mice was visualized by Western blot (Figure 2C). While fibrin(ogen) immunoreactivity was largely absent in the non-ischemic contralateral hemispheres, a strong signal was detected in the ischemic cortices which however, did not differ between the groups.

## Discussion

This is the first study examining *TAFI^-/-^* mice in an in vivo model of focal cerebral ischemia. Contrary to our initial hypothesis, *TAFI* deficiency did not protect from acute ischemic stroke.

Existing data strongly support the concept that TAFI acts as a molecular link between coagulation and fibrinolysis [18]. TAFIa restrains fibrinolysis through eliminating plasminogen binding sites from degraded fibrin and direct inhibition of plasminogen activation [8], [9]. Given this prothrombotic mechanism of TAFI action we assumed that TAFI deficiency would increase fibrinolysis and consequently, ameliorate ischemic brain damage after tMCAO in mice, a model in which microvascular thrombosis and fibrin deposition are key pathophysiological steps [16], [19], [20]. Another observation indicative for TAFI being an attractive pharmacological target to treat or prevent thromboembolic disorders is the absence of excess bleeding in *TAFI^-/-^* mice [15]. Such a safe therapy would be particularly advantageous for the treatment of acute ischemic stroke, where the conventional thrombolytics and anticoagulants used are inevitably associated with increased bleeding-related morbidity and mortality.

Whether endogenous fibrinolysis in *TAFI^-/-^* mice is indeed enhanced under pathophysiological conditions in vivo is still under debate. Nagashima and co-workers found no differences in occlusion time in TAFI-deficient mice compared to controls after either artificial arterial or venous injury [15]. Moreover, TAFI deficiency neither improved the survival rate after thrombin-induced pulmonary thromboembolism nor in an endotoxin model of disseminated intravascular coagulation [15]. In contrast, plasma clots produced from the same knockout mice lysed faster in vitro and less fibrin was retained in the lungs of TAFI-deficient mice after batroxobin-induced pulmonary embolism in another study [21]. We here further extend these findings by demonstrating that TAFI deficiency has no major beneficial effect on intracerebral fibrin formation and neuronal damage after ischemic brain challenge.

Whether our results also apply to the human situation needs further clarification. Here, plasma TAFI levels were increased in patients suffering from acute ischemic stroke and correlated with stroke severity in several retrospective studies [22]–[26]. A recent study could establish an association between the Thr325IIe polymorphism of TAFI with the incidence and age of stroke onset [27]. However, contradictory reports identifying low TAFI concentrations as a risk factor for stroke and myocardial infarction have also been published [28]–[30]. While excessive TAFI levels could foster thrombosis by inducing a hypofibrinolytic state, the inverse association between reduced TAFI concentrations and higher numbers of thrombotic events might by a consequence of enhanced inflammation since the anti-inflammatory properties of TAFI are well established [28], [31]–[33]. The finding that C-reactive protein (CRP) antigen levels parallel the course of TAFI both in the acute (increase) and later phase (decrease) of ischemic stroke further support an association between hemostasis and inflammation in this disease [25].

Only recently, the safety and efficacy of the direct thrombin inhibitor dabigatran in preventing systemic embolism including stroke in patients with atrial fibrillation has been demonstrated [34, comment in 35]. Interestingly, dagibatran enhances clot susceptibility to fibrinolysis at least partially by TAFI-dependent mechanisms in vitro [36]. In line with these findings, TAFIa significantly affected the efficacy of rt-PA and vessel recanalization in patients undergoing thrombolysis after acute ischemic stroke [37] although in vitro experiments suggested that the clot lysis rate by pharmacological concentrations of rt-PA is not altered by TAFIa [38].

In summary, despite the definite broad influence of TAFI in the coagulation-fibrinolysis system, our present study could not demonstrate a beneficial effect of TAFI deficiency on clot formation and successive neuronal degeneration after tMCAO in mice. Whether this also applies to other thrombus-dependent models of acute ischemic stroke needs to be further established.

## Materials and Methods

### Animals

A total of 38 mice were used in this study. Animal experiments were approved by legal state authorities (Bezirksregierung of Unterfranken, approval number 54-2531.01-25/06) and conducted according to the recommendations for research in basic stroke studies [39]. *TAFI^-/-^* mice were described previously [40] and backcrossed more than 10 times to C57Bl/6 background. Extensive haematological characterization of *TAFI^-/-^* mice can be found elsewhere (review in [14]). Wild-type (WT) C57Bl/6 animals were obtained from Harlan Winkelmann (Borchen, Germany) and used as controls. Male *TAFI^-/-^* and WT mice were aged 6–8 weeks.

### Murine stroke model

Focal cerebral ischemia was induced by 60 min tMCAO as described [16], [17]. Mice were anesthetized with 2.5% isoflurane (Abbott, Wiesbaden, Germany). Following a midline skin incision in the neck, the proximal common carotid artery and the external carotid artery were ligated and a standardized silicon rubber-coated 6.0 nylon monofilament (60SPPK10; Doccol Corp., Redlands, CA, USA) was inserted and advanced via the right internal carotid artery to occlude the origin of the right MCA. Operators were blinded to the genotype and operation time per animal did not exceed 15 minutes. The intraluminal suture was left in situ for 60 minutes. Then animals were re-anesthetized and the occluding monofilament was withdrawn to allow reperfusion.

The exclusion criteria were as follows:

- Death within 24 h after tMCAO
- Subarachnoid hemorrhage (as macroscopically assessed during brain sampling)
- Bederson score  =  0 (24 h after tMCAO)

Two animals (14.3%) in the wild-type group and one animal (7.7%) in the in *TAFI^-/-^* group met at least one of these exclusion criteria and were withdrawn from the study.

### Determination of infarct size and histology

Edema-corrected infarct volumes were quantified by planimetry from 2,3,5-Triphenyltetrazoliumchloride (TTC)-stained brain sections 24 h after ischemic stroke as described [16], [17]. For morphological assessment, paraffin embedded brains were stained with hematoxylin and eosin (H&E).

### Assessment of functional outcome

24 h after tMCAO the modified Bederson score [41] was used to determine global neurological function according to the following scoring system: 0, no deficit; 1, forelimb flexion; 2, decreased resistance to lateral push; 3, unidirectional circling; 4, longitudinal spinning; 5, no movement. Motor function and coordination were evaluated by the grip test [42]. For this test, the mouse was placed midway on a string between two supports and rated as follows: 0, falls off; 1, hangs onto string by one or both forepaws; 2, as for 1, and attempts to climb onto string; 3, hangs onto string by one or both forepaws plus one or both hindpaws; 4, hangs onto string by fore- and hindpaws plus tail wrapped around string; 5, escape (to the supports). Rating was performed in a blinded manner.

### Laser-Doppler flowmetry

Laser-Doppler flowmetry (Moor Instruments, Axminster, U.K.) was used in some animals (n = 4/group) to monitor rCBF in the MCA territory (6 mm lateral and 2 mm posterior from bregma) [43].

### Assessment of the cerebral vasculature

For assessment of the cerebral vasculature WT and *TAFI^-/-^* mice (n = 3/group) were deeply anesthetized with CO2 and transcardially perfused with 4% paraformaldehyde (PFA), followed by 3 ml black ink diluted in 4% PFA (1∶5 v/v). Brains were carefully removed, fixed in 4% PFA overnight at 4°C and the Circle of Willis and major arteries were examined under a microscope.

### Protein extraction and Western blot analysis

After TTC staining the cortices were dissected from formalin-fixed brain slices and homogenized in RIPA buffer (25 mM Tris pH 7.4, 150 mM NaCl, 1% NP40) containing 2% SDS. The samples were incubated for 20 minutes at 100°C followed by incubation at 60°C for 2 hours. Afterwards, tissue lysates were centrifuged at 15.000×g for 20 minutes at 4°C and supernatants were used for BCA protein assay and subsequent Western blot analysis. The total lysates were treated with SDS-PAGE loading buffer (final conc. 65 mM Tris, 5% 2-mercaptoethanol, 3% SDS, 10% glycerol) at 95°C for 5 minutes. 30 µg of total protein was electrophoresed and transferred to a PVDF membrane. After blocking for 1 h with blocking buffer (5% nonfat dry milk, 50 mM Tris-HCl pH 7.5, 150 mM NaCl, 0.05% Tween-20) membranes were incubated with the primary antibody at 4°C overnight at the following dilutions: anti-Fibrinogen pAb 1∶500 (Acris Antibodies) and anti-Actin mAb 1∶10,000 (Dianova). After a washing step with TBS-T (50 mM Tris-HCl pH 7.5, 150 mM NaCl, 0.05% Tween-20), membranes were incubated for 1 hour with HRP-conjugated donkey anti-rabbit IgG (for Fibrinogen) or donkey anti-mouse IgG (for Actin) at a dilution of 1∶5000 and were finally developed using ECLplus (GE Healthcare).

### Statistics

Data are expressed as mean ± standard deviation (SD). For statistical analysis, PrismGraph 4.0 software package (La Jolla, CA, USA) was used. Infarct volumes and neurological scores were analyzed using the non-parametric Mann Whitney test. Laser Doppler flowmetry data were compared by Bonferroni-corrected 1-way ANOVA. P-values <0.05 were considered to be statistically significant. For power and type-II (beta) error calculations on infarct volumes GraphPad Stat Mate 2.0 software package was used (GraphPad Software, Inc, La Jolla, CA, USA). The mean infarct volume at day 1 after 60 min tMCAO in C57Bl/6 WT mice was 96±27 mm^3^ and in *TAFI^-/-^* mice 91±22 mm^3^ (Fig. 2A). We assumed that a≥35% reduction (Delta = 34.0 mm^3^) of infarct size would be of biological relevance [44], [45]. The significance level (alpha) was chosen as 0.05 (two-tailed). Given those premises the power to detect a difference between means of infarct volumes of 34.0 mm^3^ was 90% in our study (type-II [beta] error: 10%) which is a favourable result compared to many other experimental stroke studies [39], [46].
